# Development of a PCR Assay to detect Papillomavirus Infection in the Snow Leopard

**DOI:** 10.1186/1746-6148-7-38

**Published:** 2011-07-18

**Authors:** Katherine Mitsouras, Erica A Faulhaber, Gordon Hui, Janis O Joslin, Curtis Eng, Margaret C Barr, Kristopher JL Irizarry

**Affiliations:** 1 College of Osteopathic Medicine of the Pacific, Western University of Health, Sciences, Pomona, CA, USA; 2 The Applied Genomics Center, Graduate College of Biomedical Sciences, Western University of Health Sciences, Pomona, CA, USA; 3 College of Veterinary Medicine, Western University of Health Sciences, Pomona, CA, USA; 4 Los Angeles Zoo and Botanical Gardens, 5333 Zoo Drive, Los Angeles, CA, USA

## Abstract

**Background:**

Papillomaviruses (PVs) are a group of small, non-encapsulated, species-specific DNA viruses that have been detected in a variety of mammalian and avian species including humans, canines and felines. PVs cause lesions in the skin and mucous membranes of the host and after persistent infection, a subset of PVs can cause tumors such as cervical malignancies and head and neck squamous cell carcinoma in humans. PVs from several species have been isolated and their genomes have been sequenced, thereby increasing our understanding of the mechanism of viral oncogenesis and allowing for the development of molecular assays for the detection of PV infection. In humans, molecular testing for PV DNA is used to identify patients with persistent infections at risk for developing cervical cancer. In felids, PVs have been isolated and sequenced from oral papillomatous lesions of several wild species including bobcats, Asian lions and snow leopards. Since a number of wild felids are endangered, PV associated disease is a concern and there is a need for molecular tools that can be used to further study papillomavirus in these species.

**Results:**

We used the sequence of the snow leopard papillomavirus UuPV1 to develop a PCR strategy to amplify viral DNA from samples obtained from captive animals. We designed primer pairs that flank the *E6 *and *E7 *viral oncogenes and amplify two DNA fragments encompassing these genes. We detected viral DNA for *E6 *and *E7 *in genomic DNA isolated from saliva, but not in paired blood samples from snow leopards. We verified the identity of these PCR products by restriction digest and DNA sequencing. The sequences of the PCR products were 100% identical to the published UuPV1 genome sequence.

**Conclusions:**

We developed a PCR assay to detect papillomavirus in snow leopards and amplified viral DNA encompassing the *E6 *and *E7 *oncogenes specifically in the saliva of animals. This assay could be utilized for the molecular investigation of papillomavirus in snow leopards using saliva, thereby allowing the detection of the virus in the anatomical site where oral papillomatous lesions develop during later stages of infection and disease development.

## Background

**Figure 1:**
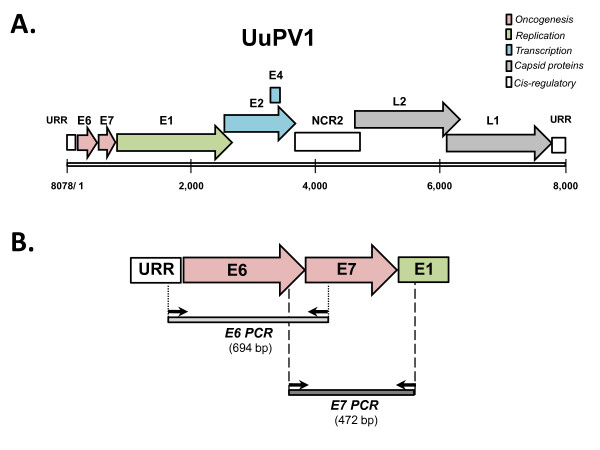
**Snow leopard PV1 genomic map and PCR strategy for detection of viral DNA**. **(a) **Schematic representation of the linear Uu PV1 genome. The numbers indicate the genomic nucleotide positions. The individual open reading frames (ORFs) of the early (E) and late (L) genomic regions are depicted as shaded arrows, and are color-coded according to the known function of the corresponding gene product. The two cis-regulatory regions are depicted as open rectangles and consist of the upstream regulatory region (URR) and the second non-coding region (NCR2). **(b) **Schematic representation of the genomic targets for PCR-based detection of viral DNA. The primers used for amplification of the *E6 *and *E7 *genes are depicted as arrows, and the resulting amplification products as shaded rectangles. The size of each amplification product is indicated.

Papillomaviruses (PVs) are a group of small, non-encapsulated epitheliotropic DNA viruses. PVs infect basal keratinocytes and cause benign proliferative lesions, termed papillomas, on the surface of cutaneous and mucosal tissues [1]. A subset of PVs are associated with the development of epithelial malignancies, such as cervical carcinomas, oral squamous cell carcinomas (OSCCs) and head and neck squamous cell carcinomas (HNSCCs) in humans [2,3]. The genomes of PVs are circular and double-stranded and contain up to 8 viral genes that are classified as early (E) or late (L) based on their temporal pattern of expression. Early genes encode regulatory proteins that function in viral replication (*E1 *and *E2*), viral shedding (*E4*), or transformation (*E5*, *E6 *and *E7*) [4], whereas late genes (*L1 *and *L2*) encode viral capsid proteins (Figure 1a) [1,5]. PVs are strictly species-specific and have been identified in a wide variety of vertebrates, including mammals, birds and recently reptiles [6,7]. Genome sequencing of several PVs and nucleotide sequence comparisons led to the development of a PV classification scheme based on *L1 *sequence identity and helped elucidate the evolutionary relationships among PVs isolated from a variety of species [7-9].

Due to their known association with human neoplasias, human papillomaviruses (HPVs) have been extensively studied, thereby increasing our understanding of the molecular mechanisms of viral oncogenesis and revealing additional factors that contribute to the development of malignancies. The finding that immune-deficient (HIV positive) or immune-suppressed (renal allograft recipients) individuals harbor higher numbers of high-risk PVs underscores the importance of immune function in the reactivation of latent infection and possibly in HPV induced oncogenesis [10-12].

Feline-specific PVs belong to the lambda-papillomavirus genus and consist of mucosal and cutaneous types [8]. In the domestic cat (*Felis domesticus*), PV infection has been associated with a number of skin lesions such as squamous cell carcinoma, bowenoid *in-situ *carcinoma, viral plaques and sarcoids [13-16]. PV associated disease is of particular interest in wild endangered felids, where these viruses have been isolated from oral papillomatous lesions in the Florida panther (*Puma concolor coryi*; PcPV1), bobcat (*Lynx rufus*; LrPV1), Asian lion (*Panthera leo persica*; PlpPV1), snow leopard (*Uncia uncia*; UuPV1) and the clouded leopard (*Neofelis nebulosa*) [9]. Sequencing and nucleotide comparisons of the LrPV1, PcPV1, PlpPV1 and UuPV1 genomes provided insight into their genomic organization, their evolutionary relationships and the mechanism of PV-host co-speciation [9]. Sequence comparisons of UuPV1 genes to those of PVs in the alpha and beta genera revealed that UuPV1 is more similar to beta-PVs (for example HPV-5) which are associated with latent infections that become reactivated under immunosuppression, than to high-risk alpha PVs (for example HPV-16), which are associated with malignancies [9].

**Figure 2:**
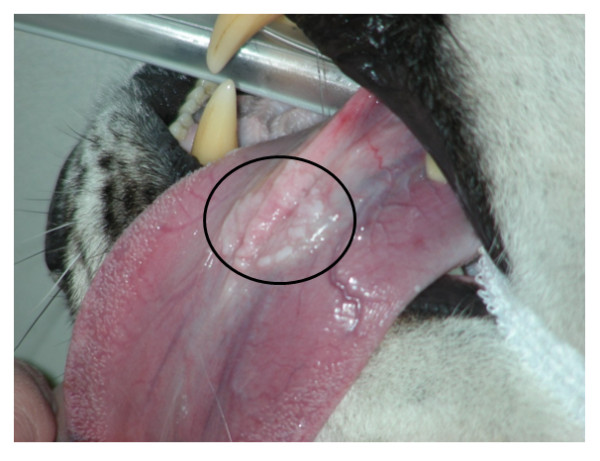
**Oral papillomatous lesions in a snow leopard**. The pale nodules on the bottom of the tongue are papillomas and are indicated by the black circle. The papillomatous lesions are arranged in a ring. This circular arrangement is likely the result of an earlier biopsy in this animal. During the biopsy, viral particles seeded the surrounding skin and caused additional papillomas to form in a circle around the original site of ablation.

Until recently, diagnosis of PV infection in snow leopards and other wild felids has depended upon observation and histopathology of papillomatous lesions. Oral papillomas in snow leopards are commonly seen under the tongue and appear as small, pale nodules (Figure 2). Very little is known about snow leopard immune responses to PV infection, and relatively few molecular tools are available to facilitate their study. Interpretation of serological tests for papillomavirus antibodies can be problematic. As with HPV infections, positive antibody tests in snow leopards probably indicate exposure but not necessarily current infection; PV antibodies may be present for several months after the virus has been cleared. False negative results are also possible because seroconversion to the major coat protein, L1, may be delayed for as much as 18 months after initiation of persistent infections, or may not occur at all [17,18]. Epithelial infections with PVs are rarely accompanied by inflammation, the viruses are non-lytic, viral capsid proteins are differentially expressed in keratinocytes and at very low levels in less mature cells, and the basal lamina of the epithelium remains intact, effectively allowing PVs to replicate without stimulating innate or adaptive immune responses [18,19]. Additionally, PVs can evade immune responses by establishing latent persistent infections, integrating into the host cell genome, or initiating a multitude of other defense mechanisms [18,20].

For endangered captive species such as the snow leopard, immune function is an important trait. In order to better understand how individual variation in snow leopard immune function relates to papillomavirus infection, appropriate molecular tools must be developed to study UuPV1 infection and its impact on the health and survival of endangered felids. In the current study, we report the development of a PCR-based assay that detects the *E6 *and *E7 *viral oncogenes in the saliva of snow leopards. This assay can be utilized as a novel molecular tool to investigate the mechanisms underlying infection and the development and progression of PV induced disease in this endangered species.

## Results

### Design of a PCR strategy to amplify papillomavirus DNA

We used the published genome sequence of UuPV1 (Figure 1a) [9] to design PCR primers that amplify viral DNA (Figure 1b). The *E6 *and *E7 *genes present ideal targets for viral DNA detection for a number of reasons. First, *E6 *and *E7 *are the key genes involved in the interaction of papillomavirus with host cell cycle pathways and are also the target of PCR-based assays that detect HPV-16 DNA in head and neck squamous cell carcinoma (HNSCC) [21], suggesting that *E6 *and *E7 *can be successfully amplified from clinical samples. Additionally, even in cases when PV integrates into the host genome and a number of viral genes are lost, *E6 *and *E7 *may be the only genes that remain intact [20]. Therefore, a PCR strategy focusing on *E6 *and *E7 *maximizes the probability of identifying viral DNA in a variety of snow leopard specimens corresponding to different stages of disease progression. We designed two sets of primers that amplify the full-length *E6 *and *E7 *genes as two separate PCR products (Figure 1b). The two primer pairs are positioned such that the *E6 *and *E7 *PCR products overlap by 95 nucleotides, which allows us to confirm DNA sequence information from two independent PCR products as an additional internal control. The *E6 *primers anneal at genomic positions 12 (left primer) and 705 (right primer), and amplify a 694-bp fragment encompassing the entire *E6 *coding region (which spans genomic positions 31-447), and the first 267 bp of the *E7 *gene (located at genomic positions 444-728). The *E7 *primers anneal at genomic positions 327 (left primer) and 800 (right primer) and produce a 472-bp fragment, that contains the last 120 bp of the *E6 *gene and the entire *E7 *coding region.

### PCR amplification of viral DNA from snow leopard saliva

**Figure 3:**
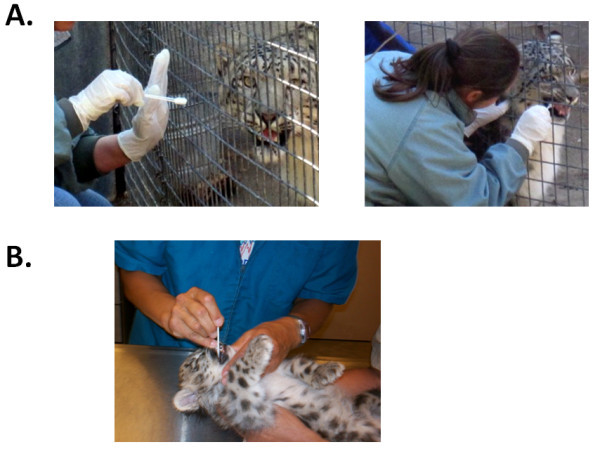
**Collection of saliva samples from a captive snow leopard using the Oragene •ANIMAL kit**. **(a) **Saliva was collected from a 2-year old female snow leopard in its enclosure using the Oragene •ANIMAL kit. The sample was collected by the handler of the animal through the cage using two Oragene •ANIMAL sponges, and was stabilized in the Oragene •ANIMAL solution. **(b) **Saliva collection using the Oragene •ANIMAL kit from a 7-week old female cub during a routine veterinary exam.

Because papillomatous lesions occur within the snow leopard oral cavity [9,22], we wanted to determine whether saliva represents an appropriate biological specimen for the detection of papillomavirus DNA in asymptomatic animals. Additionally, since saliva collection can be performed in a non-invasive manner, behavioral modification techniques can be used to train animals to calmly accept an oral swab without the need for chemical restraint. In this fashion, we were able to obtain saliva samples from snow leopards in their enclosure, thus minimizing anxiety to the animals and without introducing them into stress-provoking situations (Figure 3a). This is particularly important for captive species that are not routinely subjected to chemical restraint, and are only anesthetized for scheduled physical exams every year or every two or three years. In these cases, saliva represents a more easily and frequently accessible biological specimen than blood.

**Figure 4:**
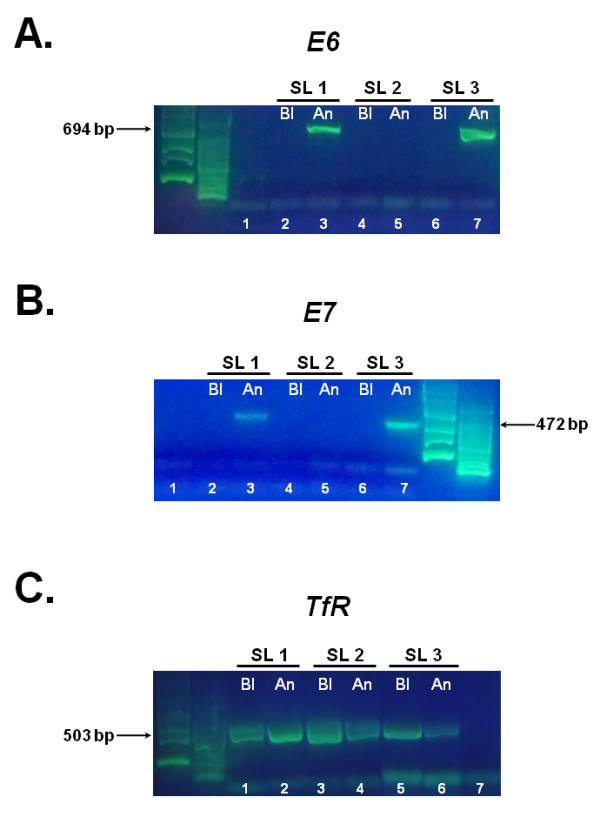
**Amplification of the UuPV1 *E6 *and *E7 *genes from paired saliva and blood samples**. 50 ng of genomic DNA isolated using the Oragene•ANIMAL kit (An), or paired blood samples (Bl) from three snow leopards (SL 1, 2 and 3) were used for PCR amplification. Amplification products were resolved by agarose gel electrophoresis and visualized by staining with SYBR^® ^Green. **(a) **Amplification of a 694-bp fragment encompassing the *E6 *viral oncogene from saliva (lanes 3, 5 and 7) and blood (lanes 2, 4 and 6). An amplification control with no DNA was performed in parallel (lane 1). **(b) **Amplification of a 472-bp DNA fragment encompassing the *E7 *viral oncogene from paired saliva (lanes 3, 5 and 7) and blood samples (Bl; lanes 2, 4 and 6). Lane 1 shows a control amplification reaction with no DNA. **(c) **Control amplification reactions of a 503-bp fragment in the coding region the snow leopard Transferrin Receptor (TfR) gene, using paired saliva (An; lanes 2, 4 and 6) and blood samples (Bl; lanes 1, 3 and 5). The negative amplification control with no DNA is shown in lane 7.

A positive result in a PCR-based assay would indicate presence of the virus in the oral cavity of the animal, as opposed to a serological test which can indicate exposure, but not necessarily current infection. We obtained matched saliva and blood samples from three snow leopards that did not have oral papillomatous lesions: two adult females that were approximately 18 years old (SL 1 and SL 3), and a 7-week old female cub (SL 2; Figure 3b). We used the *E6 *and *E7 *primer pairs in amplification reactions using DNA purified from the paired blood and saliva samples (Figure 4a and 4b). We obtained amplification products of the expected size for both *E6 *(Figure 4a, lanes 3 and 7) and *E7 *(Figure 4b, lanes 3 and 7) using saliva from the two adult snow leopards (SL 1 and SL 3) but not from the cub (Figure 4a, lane 5; Figure 4b lane 5). Additionally, we were unable to detect viral DNA for *E6 *and *E7 *in DNA purified from the matched blood samples of the two adult snow leopards (Figure 4a, lanes 2 and 6; Figure 4b, lanes 2 and 6). This was not due to the absence of DNA in any of the saliva or blood samples, since we successfully amplified a 503-bp product corresponding to the coding region of the snow leopard transferrin receptor (TfR) as a positive control (Figure 4c, lanes 1-6). Additional saliva samples obtained from two snow leopards at another facility were negative for *E6 *and *E7 *DNA (data not shown).

### Validation of the E6 and E7 PCR products by restriction digest and DNA sequencing

**Figure 5:**
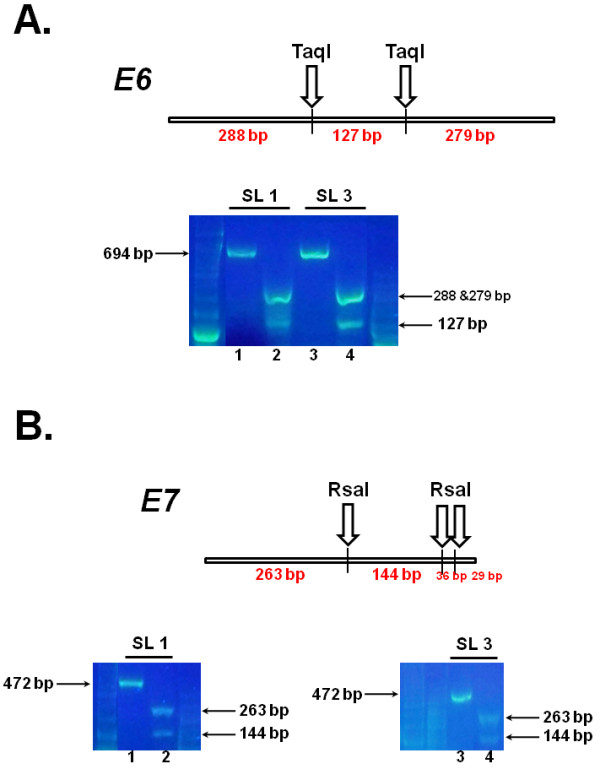
**Validation of the *E6 *and *E7 *amplification products by restriction enzyme digestion**. **(a) **Top panel: Schematic representation of the 694-bp *E6 *amplification product indicating the positions of the two TaqI cleavage sites, and the sizes of the resulting fragments (288, 279 and127 bp). Bottom panel: 50 ng of genomic DNA isolated from two snow leopards (SL 1 and SL 3) using the Oragene•ANIMAL kit were amplified using the *E6 *primer pair. Half of each PCR reaction was incubated with the TaqI restriction endonuclease. Restriction products (lanes 2 and 4), along with an aliquot of the original PCR reactions (lanes 1 and 3) were resolved by agarose gel electrophoresis and visualized by staining with SYBR^® ^Green. **(b) **Top panel: Schematic representation of 472-bp *E7 *amplification product indicating the positions of the three RsaI cleavage sites, and the sizes of the resulting restriction products (263, 144, 36 and 29 bp). Bottom panel: 50 ng of genomic DNA isolated from two snow leopards (SL 1 and SL 3) using the Oragene•ANIMAL kit were amplified using the *E7 *primer pair. Half of each PCR reaction was incubated with the TaqI restriction endonuclease. Restriction products (lanes 2 and 4), along with an aliquot of the original PCR reactions (lanes 1 and 3) were resolved by agarose gel electrophoresis and visualized by staining with SYBR^® ^Green.

Since restriction digests provide a fast and cost-effective method to rapidly screen PCR products, we developed a restriction endonuclease digestion assay of the amplification products to confirm that they correspond to the *E6 *and *E7 *viral genes. The restriction enzyme TaqI cleaves the *E6 *PCR product twice, producing three fragments of 288, 279 and 127 bp (Figure 5a, top panel). The *E7 *PCR product contains three RsaI restriction sites, yielding two large fragments (263 and 144 bp) and two smaller fragments (36 and 29 bp; Figure 5b, top panel). Digestion of the *E6 *PCR products obtained from the saliva of the two older snow leopards (SL 1 and SL 3) produced a doublet corresponding to the 288 and 279-bp fragments and a single 127-bp fragment (Figure 5a, bottom panel, lanes 2 and 4). Similarly, incubation of the *E7 *amplification products from these animals with RsaI yielded two fragments of the expected size (Figure 5b, bottom panels, lanes 2 and 4).

**Figure 6:**
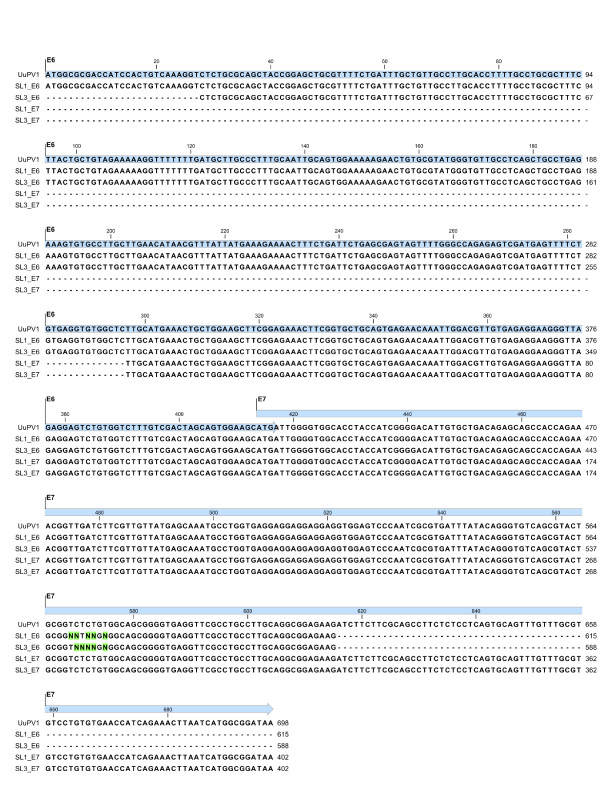
**Sequence comparison of the *E6 *and *E7 *amplification products and UuPV1 genome**. The *E6 *(SL1_E6 and SL3_E6) and *E7 *(SL1_E7 and SL3_E7) amplification products obtained from the saliva of two snow leopards were gel-purified and subjected to bi-directional Sanger sequencing. The resulting sequences were aligned to the consensus UuPV1 genome (UuPV1, Genbank: DQ180494) using CLC Sequence Viewer 6. The sequences of the *E6 *and *E7 *genes in the UuPV1 genome are highlighted in cyan and the sequence length of each amplification product is indicated. Ambiguous nucleotides in sequencing reactions are designated by Ns. Nucleotides that do not match the UuPV1 genome sequence are highlighted in green.

As an independent measure to confirm their identity, we gel purified the *E6 *and *E7 *PCR products from the two animals and subjected them to bi-directional DNA sequencing. The sequencing results were assembled into contigs and aligned to the consensus genome sequence of UuPV1 (Figure 6). The multiple sequence alignment in Figure 6 shows that the sequence of the *E6 *and *E7 *amplification products obtained from saliva is 100% identical to the published UuPV1 genome sequence for *E6 *and *E7*.

These two validation methods independently confirm the identity of the *E6 *and *E7 *amplicons. Although amplicon sequencing is preferable, it is can be more expensive, time-consuming and requires significant amounts of purified PCR products, which might not always be feasible. In such cases, restriction digests provide an accurate, simple and cost-effective method to rapidly screen even large numbers of samples. The choice of two distinct validation methods in our assay provides flexibility and makes it amenable to perform in a variety of laboratory settings.

## Discussion

We developed a molecular assay that detects papillomavirus DNA in snow leopard saliva. Our method is similar to PCR-based molecular assays designed to detect different types of human papillomavirus (HPV) in a variety of clinical samples [21,23]. Specifically, presence of HPV-16 in head and neck squamous cell carcinoma (HNSCC) biopsies is determined by amplification of the *E6 *and *E7 *genes [21]. Similarly, *E6 *amplification is used to detect the presence of high-risk HPV subtypes in human cutaneous neoplasms and in cervical smears [23-25]. In some cases of PV integration into host genetic material, *E6 *and *E7 *may be the only genes that remain intact [20], thus making them ideal choices for PCR-based detection of viral DNA.

The detection of UuPV1 DNA in the saliva of two older snow leopards parallels results from HPV studies that have demonstrated that HPV DNA can be amplified from oral rinses and saliva samples from HNSCC patients and HIV positive individuals [10,26-28]. Because PVs are epitheliotropic, detection of PV DNA in blood samples from infected snow leopards is unlikely [18]; therefore, the absence of viral DNA in blood is consistent with an expected PV replication pattern in mucosal cells with no concurrent viremia.

The utilization of a PCR-based method to detect viral DNA in saliva presents a number of advantages. First, saliva collection is a non-invasive sampling method, thereby circumventing the requirement for anesthesia prior to obtaining a blood sample in captive wild felids. This is particularly important for captive species that are not routinely subjected to chemical restraint, and may only be anesthetized for scheduled physical exams. In these cases, saliva represents a more easily and frequently accessible biological specimen than blood. An additional advantage of testing saliva is that behavioral modification techniques can be used to train snow leopards and other felids to calmly accept an oral swab for DNA collection without chemical restraint. In fact, we were able to collect saliva samples from snow leopards in their enclosure, with minimal disturbance to the animal. Subsequently, animals testing positive for viral DNA can then be scheduled for a more thorough oral examination under sedation. The use of saliva also allows for detection of the virus in the anatomical site where papillomatous lesions develop during later stages of disease progression. Additionally, saliva is extremely stable, and more easily preserved than serum samples, thereby facilitating collection and shipping of samples from different sites. In testing for HPV, it has been demonstrated that PCR-based methods have increased sensitivity as compared to serotesting [29], and can accommodate even lower quality samples, such as archival samples and paraffin-embedded specimens [23]. In contrast to seropositivity which can indicate a present and ongoing infection or a past infection that has been cleared, a positive PCR test indicates a current infection in the oral cavity of the animal. Therefore, PCR-based testing of snow leopard saliva can complement serotesting and help provide a more accurate picture of an animal's disease status.

PCR-based detection of papillomavirus *E6 *and *E7 *DNA in the saliva of snow leopards may have utility in diagnosing infection in animals that have no obvious oral papillomatous lesions, confirming infection in cats with oral papillomas, and detecting infection in animals that have not yet seroconverted. Due to its non-invasiveness and ease of implementation, PCR-based testing of saliva can be incorporated into the routine and regular screening of captive snow leopards, which would greatly facilitate the collection of epidemiological data on UuPV1 infections. Additionally, since papillomatous lesions have been reported to progress to SCC in snow leopards [9,22], our assay can also be used to determine whether viral DNA is present in biopsy samples of SCCs to explore the relationship between UuPV1 infection and neoplasia and elucidate the molecular mechanisms underlying the development and progression of PV induced disease.

The finding that UuPV1 DNA is present in the saliva of two adult snow leopards that have no current or documented lesions is intriguing. The most likely explanation for our finding is supported by the advanced age of these animals and the fact that saliva was collected following euthanasia due to progressive illness. Papillomaviruses can establish latent or low-level inapparent infections, remaining undetected for long periods of time [1], and these cats may have been experiencing reactivation of a previously controlled PV infection. It is also possible, although unlikely, that these animals had recently become infected, and PV induced disease was not manifested at the time of death.

One of the goals of conservation biology is to maintain genetically diverse captive populations of endangered species. In relatively small populations, like the 150-200 captive snow leopards in North America, it is critical to develop functional assays for assessing individual differences in health and fitness. One such difference is an animal's susceptibility to PV infection and to the development of PV associated disease. In the current study, we demonstrate that viral DNA is present in the saliva of clinically normal animals. Therefore, our assay can be used to classify clinically normal snow leopards into PV-positive and PV-negative populations, which, along with clinical data, can be studied to identify both genetic and environmental factors underlying susceptibility to viral infection and to the development and progression of disease. Similar studies in humans have identified specific variants in immune genes as well as in genes that interact with viral proteins and demonstrated that these variants are associated with an individual's risk for viral persistence and with the subsequent development of disease [30,31].

The conservation of endangered captive species requires maintaining the genetic diversity of the population. When captive populations suffer from inbreeding depression, fitness decreases and mortality rates may increase along with decreased reproductive success and impaired immune function [32,33]. Genetic studies in small populations of endangered species are challenging because standard approaches like large-scale genetic association studies are not possible. Instead, genetic approaches must focus on individuals and place the emphasis on developing effective means of characterizing phenotypes which can be used to study phenotypic variation across the captive population. In the case of the snow leopard, the well-documented infection of animals with papillomavirus [22] offers an opportunity to develop robust phenotyping methods for assessing immune function within the captive population. Unfortunately, relatively little is known about snow leopard susceptibility and resistance to papillomavirus infection and disease progression. In order to better understand the potential phenotypic variation underlying susceptibility and resistance to viral infection, it is critical that molecular tools be developed that can allow investigators to begin characterizing how phenotypes associated with papilomavirus infection and disease progression vary across closely and distantly related individuals. The PCR assay we describe is a first step in this direction. This assay can be used to (1) monitor the exposure of individual snow leopards to papillomavirus, (2) accurately identify which snow leopards are positive and which are negative for papillomavirus, (3) characterize the relationship between papillomavirus exposure and the development of oral papillomatous lesions, (4) investigate the relationship between exposure and infection when uninfected snow leopards are housed with infected snow leopards and, (5) determine the timeline underlying subclinical infection and presentation of clinical signs. As more snow leopards participate in these studies, distributions describing these traits for the entire captive population can be produced. Subsequently, individuals exhibiting phenotypes at the tails of the distributions can be identified and further studies aimed at characterizing the genetic basis of snow leopard immunity in captivity can be performed.

## Conclusions

We developed a novel PCR strategy to detect papillomavirus in the snow leopard. Using this assay, we successfully amplified the *E6 *and *E7 *viral oncogenes in DNA purified from saliva of two snow leopards. DNA sequencing verified that the amplified fragments indeed represent the *E6 *and *E7 *genes of UuPV1. In addition, we demonstrated that viral DNA cannot be detected in paired blood samples from these animals, which is consistent with the mechanism of papillomavirus infection and viral lifecycle in the host. Taken together with the non-invasiveness, and ease of collection relative to blood, our results further underscore the utility of saliva as a suitable clinical specimen for the detection of papillomavirus in snow leopards. Our findings allow for the development of a molecular tool to elucidate the mechanisms underlying the development and progression of PV induced disease in this endangered species.

## Methods

### Sample collection and purification

We obtained paired saliva and blood samples from 3 captive snow leopards housed in North American zoos: two female snow leopards that were approximately 18 years old, and a 7-week old female cub. None of these animals had oral papillomatous lesions. We additionally collected saliva samples from a 2-year old female and a 2-year old male snow leopard in their enclosures without the use of anesthesia. Collection protocols were approved by the Western University Institutional Animal and Care Use Committee. Blood was drawn into PAXgene Blood DNA tubes (Qiagen, Valencia, CA, USA) and genomic DNA was isolated using the PAXgene Blood DNA kit (Qiagen, Valencia, CA, USA). Saliva was collected using Oragene•ANIMAL kits (DNA Genotek, Ontario, Canada) and DNA was purified from the entire saliva sample using the manufacturer's protocol [34]. All DNA samples were quantitated using a Nanovue spectrophotometer (GE LifeSciences, Piscataway, NJ, USA). The purity of each DNA sample was assessed using the A260/A280 ratio.

### Primer design

The published UuPV1 genome sequence [Genbank: DQ180494] was used to design PCR primers that amplify DNA fragments encompassing the entire *E6 *and *E7 *genes as shown in Figure 1(b). Primers were designed using the freely available Primer3 software package [35], and tested by In-silico PCR [36] to assess whether they non-specifically amplify feline genomic sequences. Primer sequences are as follows: E6-Forward 5'-AGTGACTCGGAGGGCATTC-3', E6-Reverse 5'-GATGGTTCACACAGGACACG-3', E7-Forward 5'-TTGCATGAAACTGCTGGAAG-3', E7-Reverse 5'-GGTTCGTCATCATCGCTACA-3'. The feline transferrin receptor (TfR) mRNA sequence [Genbank: NM_001009312] was used to design PCR primers that amplify a 503-bp fragment in the coding region of the gene. Primers were designed as described above and used for cross-species amplification of the corresponding region of the snow leopard transferrin receptor gene. Primer sequences are as follows: TfR-Forward 5'-TTTCTTGATATTTGAGTTCATTGTTT-3', TfR-Reverse 5'-AGTAACTGTCGCTGCTTTACTGT-3'

### PCR amplification

DNA extracted from the 3 sets of matched saliva and blood samples was used for amplification of the *E6 *and *E7 *genes of the snow leopard PV1. A 694-bp fragment encompassing the *E6 *gene was amplified using the E6-Forward and E6-Reverse primers. Reactions were performed in a 50 microliter volume using 50 ng genomic DNA, 0.2 μM each primer, 0.125 mM dNTPs, 1.5 mM MgCl_2_, 1X GeneAmp^® ^PCR Gold Buffer and 2.5 U Amplitaq Gold^® ^DNA Polymerase (Applied Biosystems, Foster City, CA, USA) in a Veriti™ 96-well thermal cycler (Applied Biosystems, Foster City, CA, USA) using the following conditions: 95°C for 10 min, 10 cycles of 95°C 30 sec, 1 min annealing with a starting temperature of 63°C decreasing by 0.5°C per cycle down to 58.5°C and 72°C 1 min, followed by an additional 25 cycles of 95°C 30 sec, 58°C 1 min, 72°C 1 min and a final extension for 10 min at 72°C. A 472-bp fragment encompassing the *E7 *gene was amplified using the E7-Forward and E7-Reverse primers. Amplification reactions were performed in a 50 microliter volume using 50 ng genomic DNA, 0.2 μM each primer, 0.125 mM dNTPs, 2.5 mM MgCl_2_, 1X GeneAmp^® ^PCR Gold Buffer and 2.5 U Amplitaq Gold^® ^DNA Polymerase (Applied Biosystems, Foster City, CA, USA) in a Veriti™ 96-well thermal cycler (Applied Biosystems, Foster City, CA, USA) using the following conditions: 95°C for 10 min, 12 cycles of 95°C 30 sec, 1 min annealing with a starting temperature of 61°C decreasing by 0.5°C per cycle down to 55.5°C and 72°C 1 min, followed by an additional 25 cycles of 95°C 30 sec, 55°C 1 min, 72°C 1 min and a final extension for 10 min at 72°C. 25 microliters of each PCR reaction were resolved on a 1.5% agarose/1X TBE gel stained with SYBR^® ^Green (Invitrogen, Carlsbad, CA, USA). All PCR reactions were repeated at least twice in independent experiments.

A 503-bp fragment in the coding region of the snow leopard transferrin receptor gene was amplified using the TfR-Forward and TfR-Reverse primers. Reactions were performed in a 50 microliter volume using 50 ng genomic DNA, 0.4 μM each primer, 0.25 mM dNTPs, 2.5 mM MgCl_2 _and 2.5 U Taq DNA Polymerase (Qiagen, Valencia, CA, USA) in a Veriti™ 96-well thermal cycler (Applied Biosystems, Foster City, CA, USA) using the following conditions: 95°C for 10 min, 30 cycles of 95°C 30 sec, 54°C 1 min, 72°C 1 min and a final extension for 10 min at 72°C. 15 microliters of each PCR reaction were resolved on a 1.5% agarose/1X TBE gel stained with SYBR^® ^Green (Invitrogen, Carlsbad, CA, USA). All PCR reactions were repeated at least twice in independent experiments.

### Restriction analysis

The identity of the *E6 *and *E7 *amplification products was validated by restriction enzyme digest. 25 microliters of the *E6 *amplification products obtained from two snow leopard saliva samples were incubated with 15 U TaqI (Fermentas, Glen Burnie, MD, USA) in a 30 microliter reaction at 65°C for 2 hours. Digestion products were resolved on a 2.5% agarose/1X TBE gel stained with SYBR^® ^Green (Invitrogen, Carlsbad, CA, USA). 25 microliters of each *E7 *amplification product were digested with 15 U RsaI (Fermentas, Glen Burnie, MD, USA) in a 30 microliter reaction at 37°C for 2 hours. Digestion products were resolved on a 2.5% agarose/1X TBE gel with SYBR^® ^Green (Invitrogen, Carlsbad, CA, USA).

### DNA sequencing

The *E6 *and *E7 *amplification products obtained from two snow leopard saliva samples were purified using the Qiaex II Gel Extraction kit (Qiagen, Valencia, CA, USA) and subjected to bi-directional DNA sequencing using the *E6 *and *E7 *PCR primer pairs respectively. Sequencing was performed at the UCLA Sequencing Core (Los Angeles, CA, USA). The forward and reverse sequences of each amplification product were assembled into a single contig, and aligned to the consensus UuPV1 genome sequence [Genbank: DQ180494] using the CLC Sequence Viewer 6 software package (CLC Bio, Cambridge, MA, USA).

## List of abbreviations

Bp: Basepair; DNA: Deoxyribonucleic acid; HPV: Human papillomavirus; HNSCC: Head and neck squamous cell carcinoma; HIV: Human immunodeficiency virus; OSCC: Oral squamous cell carcinoma; ORF: Open reading frame; PV: Papillomavirus; PCR: Polymerase chain reaction; TfR: Transferrin receptor.

## Authors' contributions

KM conceived of the study, participated in study design, assisted with the collection of saliva and blood samples, purified saliva and blood samples, performed PCR amplification, restriction digests and gel purification of *E6 *and *E7 *amplicons for sequencing, performed data analysis, prepared figures and drafted the manuscript. EAF participated in the design of the PCR amplification strategy, assisted with PCR amplification and drafted the manuscript. GH performed computational analysis of papillomavirus-host interactions to specifically select *E6 *and *E7 *as suitable targets for PCR-based detection of viral DNA. JOJ assisted with the clinical interpretation of oral papillomatous lesions in snow leopards. CE participated in study design and provided clinical interpretation/relevance of experimental results. MCB obtained IACUC approval, coordinated data collection, provided access to snow leopard saliva and blood samples, assisted with data interpretation and drafted the manuscript. KJLI conceived of the study, designed the study, coordinated data collection, designed the PCR amplification strategy, performed data analysis and drafted the manuscript. All authors have read and approved the manuscript.
